# Cancer incidence in Khyber Pakhtunkhwa, Pakistan, 2020

**DOI:** 10.1186/s12889-023-16686-5

**Published:** 2023-09-14

**Authors:** Farhana Badar, Muhammad Sohaib, Shahid Mahmood, Omar Rasheed Chughtai, Faisal Sultan, Muhammed Aasim Yusuf

**Affiliations:** 1 Cancer Registry and Clinical Data Management, Shaukat Khanum Memorial Cancer Hospital and Research Centre, 7-A, Block R-3, M. A. Johar Town, Lahore, 54782 Pakistan; 2 Nuclear Medicine & Oncology Division, Pakistan Atomic Energy Commission, Islamabad, Pakistan; 3 Chughtai Lab, Lahore, Pakistan; 4 Shaukat Khanum Memorial Cancer Hospital and Research Centre, Lahore, Pakistan

**Keywords:** Cancer incidence, Malignancies, Population-based, Khyber Pakhtunkhwa, Pakistan

## Abstract

**Background:**

To present the population-based cancer statistics for Khyber Pakhtunkhwa (KP), Pakistan, an incidence study was conducted at the Shaukat Khanum Memorial Cancer Hospital and Research Centre (SKMCH&RC) in Lahore, Pakistan, in 2023.

**Methods:**

Records from various centres on new cancers diagnosed among residents of KP between January and December 2020 were gathered. Both active and passive methods of data collection were applied, and the information was saved in a central repository at SKMCH&RC. The incidence rates were computed by age group and sex and presented per 100,000 population.

**Results:**

Among children (0–14 years), the Age-Standardised Incidence Rate (ASIR) was 4.0 in girls and 6.1 in boys, and haematologic malignancies were more prevalent; in adolescents (15–19 years), the ASIR was 7.7 in females, 9.4 in males, and bone tumours, haematologic malignancies, and neurological cancers were prominent; in adult females (> / = 20 years), the ASIR was 84.9, and cancers of the breast, digestive system, and reproductive organs were predominant; and adult males, the ASIR was 73.0, and cancers of the gastrointestinal tract, lip/oral cavity/pharynx, prostate, and Non-Hodgkin Lymphoma (NHL) were common.

**Conclusions:**

It is crucial to investigate the aetiology of these diseases at the community level because dietary elements, infectious diseases, and tobacco use all appear to be significant contributors. Prospective studies could play a key role in highlighting the factors linked to these diseases. Therefore, cancer registration must continue in conjunction with the exploration of risk factors.

## Background

Health systems are plagued by inequalities the world over. The need for healthcare is expanding in parallel with the population of various countries. Changes in biological, environmental, and social factors are causing new diseases and increasing disparities in access to healthcare. Global problems, such as conflict and natural disasters, as well as climate change, magnify healthcare inequalities. Recently, national health systems and practitioners have had to shift strategies during the COVID-19 pandemic, sometimes with mixed success. Most countries with mature healthcare systems also have established systems for cancer surveillance. In less developed countries, those systems that do exist tend to be unsynchronized, reflecting a lack of coordination among stakeholders. Examples of such initiatives in Pakistan are the Punjab Cancer Registry and the Karachi Cancer Registry in the east and south of the country, respectively [1, 2]. Coherence is important to ensure maximum gains from such standalone efforts.

According to estimates from Globocan, 19.3 million new cancer cases were reported globally in 2020 [3], with 3.5 million of those occurring in low- and middle-income countries, which accounted for about one-fifth of cancer cases worldwide [4, 5]. In Pakistan, over 178,000 new cancer cases were estimated in 2020, with 117,149 deaths in an ethnically diverse population of 220 million people [6]. These deaths accounted for 2% of all cancer-related deaths (9.9 million) worldwide [3].

As already mentioned, Pakistan has a limited number of operational registries in the east and south of the country. The results from these registries state that among children and adolescents, bone tumours and leukaemia are relatively common, whereas, among adult women, malignancies of the breast, reproductive organs, colorectum, lip/oral cavity/pharynx, and liver, and among adult men, cancers of the prostate, bladder, respiratory tract, lip/oral cavity/pharynx, and liver have relatively high incidence rates. It is comparable with Globocan’s projections, which list cancers of the breast, lip/oral cavity, lung, oesophagus, and colorectum as the most frequently reported cancers in both sexes combined.

Population-level statistics for Pakistan have recently been reported [7]. The initial report states that malignancies in KP accounted for 16.5% of the cases in the country between 2015 and 2019 [7]. As a separate endeavour, systematic data collection for cancer in the northwest region of Pakistan started in 2017. In this manuscript, we report incidence rates for cancers diagnosed in the province of Khyber Pakhtunkhwa. According to the preliminary assessment, the top ten malignancies in the northwest region are more similar than different from those found in the east and south of the country.

## Methods

### The catchment area

**Fig. 1:**
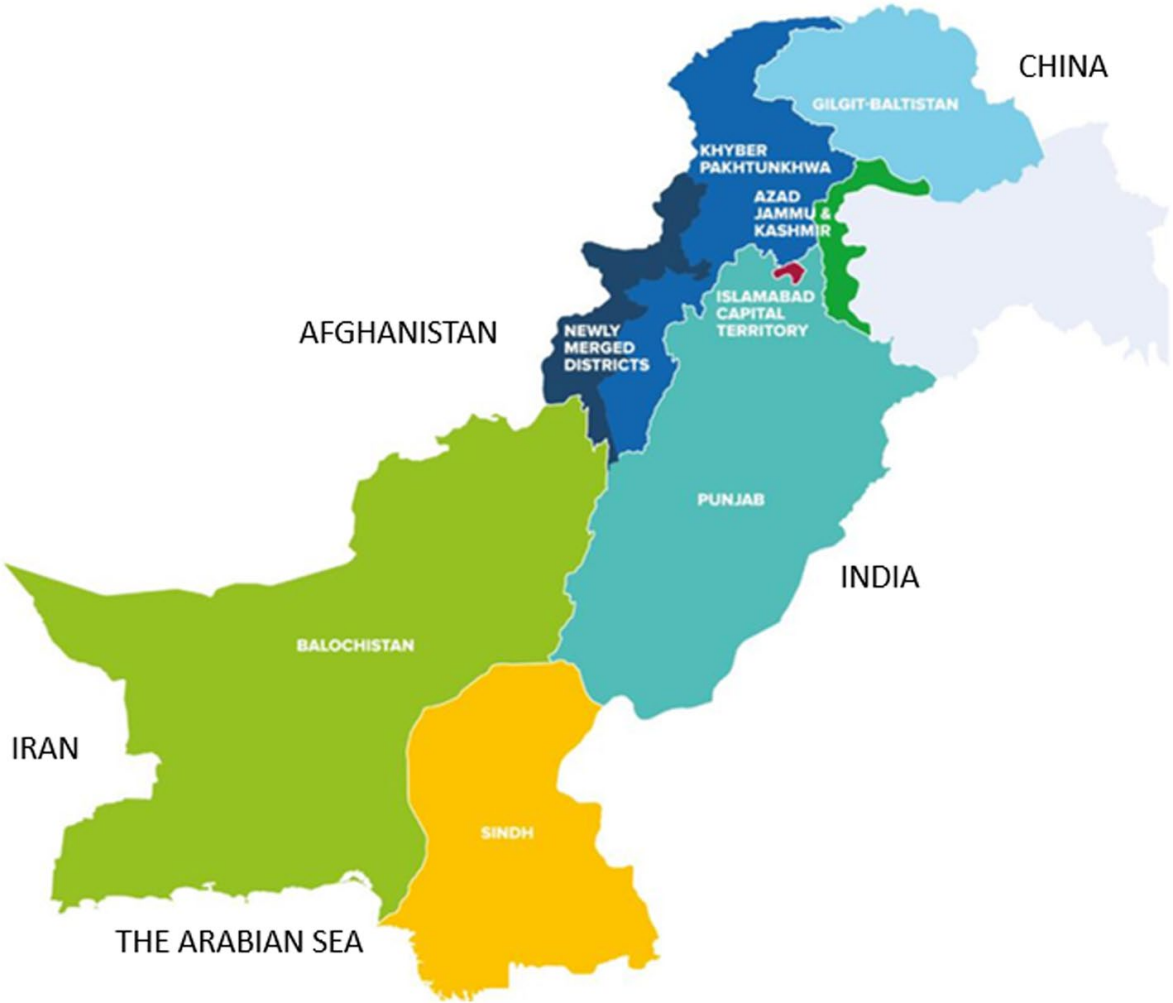
Map of Pakistan showing the provinces and regions adjacent to Pakistan (The royal and navy blue colours stand for Khyber Pakhtunkhwa. The names of the territories that border Pakistan have been inserted; they were not present in the original map)

The province of KP is in the northwest part of the country, as shown in the map (Fig. 1) [8]. It shares borders with Afghanistan to the west, Balochistan to the south, Punjab to the southeast and east, Azad Jammu and Kashmir to the northeast, and Gilgit-Baltistan to the north. The Federally Administered Tribal Areas (FATA) are a part of KP as of 2018 [9]. Based on the Census Bureau’s 2017 report and average annual growth rates of 2.89% for KP and 2.40% for FATA, the combined KP had a population of 38,589,937 people in 2020 (KP 33,229,047 and FATA 5,360,890), with a population density of 592.83 people per square kilometre (KP 408.40 and FATA 183.43) over an area of 101,741 square kilometres; nearly 20% of the population lived in urban areas [9, 10]. Hereon, KP refers to the province inclusive of FATA.

The major ethnic groups in the province are the Pashtuns (Pathans). Other smaller ethnic groups include the Hindkowans, Dards, Chitralis, Kalashis, and Gujjars [11].

### Data collection

Information on new cancers diagnosed in the KP population over a year, from January 1, 2020, to December 31, 2020, was collected retrospectively from the dataset and database available in the Cancer Registry and Clinical Data Management section at SKMCH&RC. The information provided by the Pakistan Atomic Energy Commission (PAEC) Cancer Hospitals, SKMCH&RC, and Chughtai Lab was reviewed and analysed. It is noteworthy that five of the nineteen cancer hospitals PAEC operates nationwide are in KP [12]. SKMCH&RC operates two hospitals in Pakistan, one in Peshawar, KP, and the other in Lahore, Punjab [13]. There are 176 sample collection centres run by SKMCH&RC across the country, twenty-four of which are in KP. Twenty-six of the 318 collection facilities operated by Chughtai Lab are in KP [14]. Our review did not include patients from Afghanistan who provided an address within Afghanistan.

The most valid basis for cancer diagnosis was histology, cytology, specific tumour markers, clinical investigation, or clinical diagnosis. Many cases were histologically verified. Cancer cases were coded using the International Classification of Diseases for Oncology, third edition (ICD-O-3) coding systems [15]. These were further categorised using the International Classification of Diseases, Tenth Revision, Clinical Modification (ICD-10-CM) [16, 17]. Multiple primaries were managed per international standards [15]. All malignancies with a behaviour code of /2 (in-situ) or /3 (malignant) were included. Central Nervous System (CNS) tumours (brain and nervous system), with a behaviour code of /0 (benign) and /1 (borderline malignancy) were also included [15]. Duplicate entries were removed by performing an edit check. Data validity was examined for site versus histology and gender versus site and histology. Cancer counts, crude incidence rates and ASIRs were calculated and presented per 100,000 people by sex and age group (children, young adults/adolescents, and adults). Segi’s world standard population was applied to standardise the rates [18]. The rates were computed using Microsoft Excel 2016, which was also used to make tables and graphs.

## Results

**Fig. 2:**
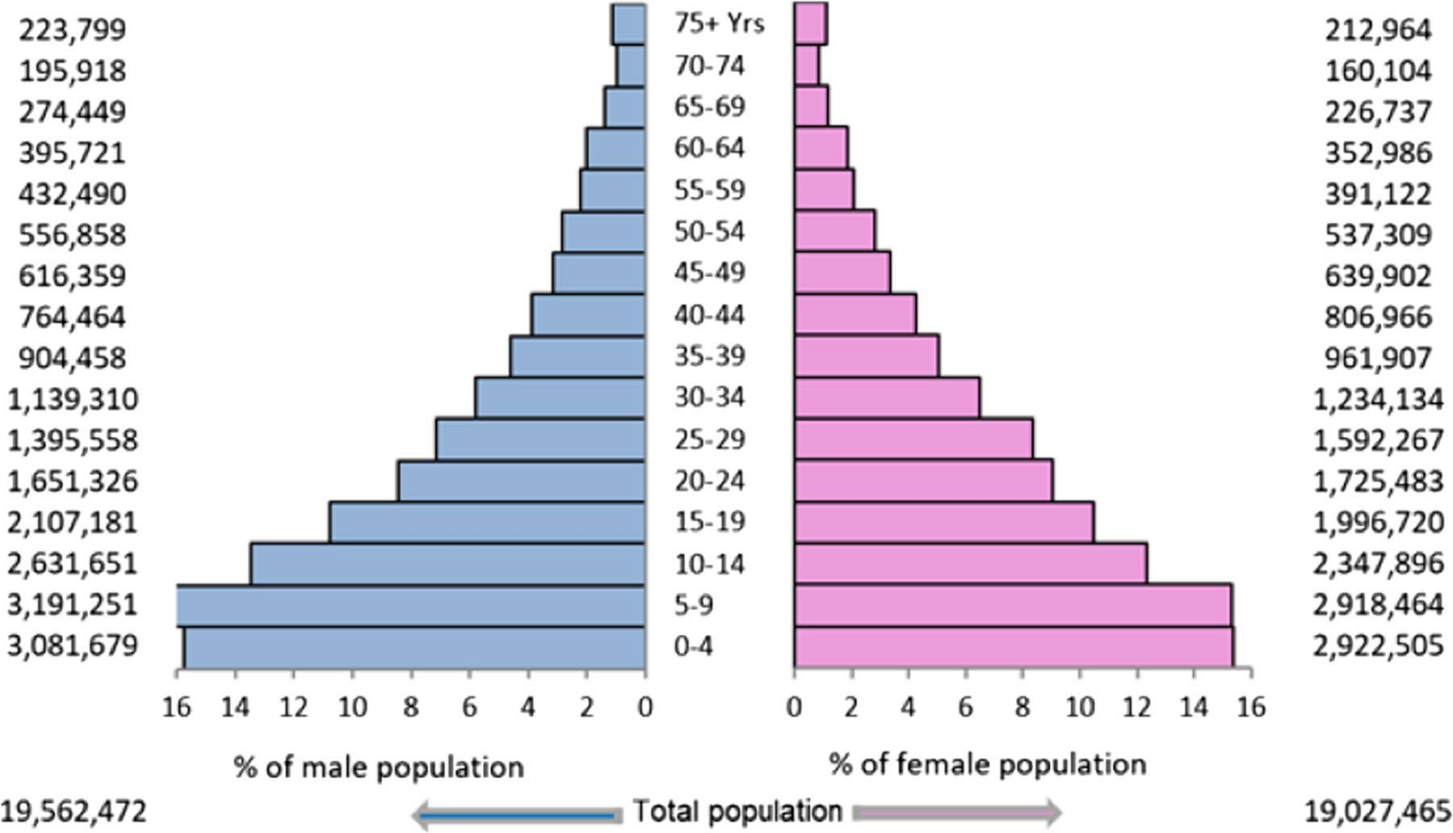
Population pyramid showing average annual person-years by sex and age group in Khyber Pakhtunkhwa, Pakistan, 2020

**Fig. 3:**
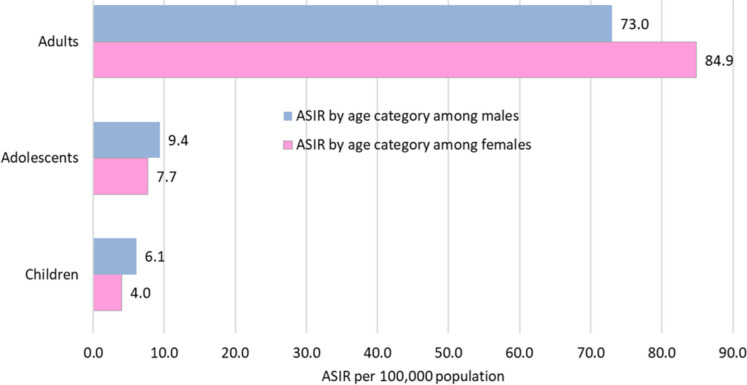
Age-Standardised Incidence Rates (ASIR) per 100,000 population by sex and age group, Khyber Pakhtunkhwa, Pakistan, 2020

**Fig. 4:**
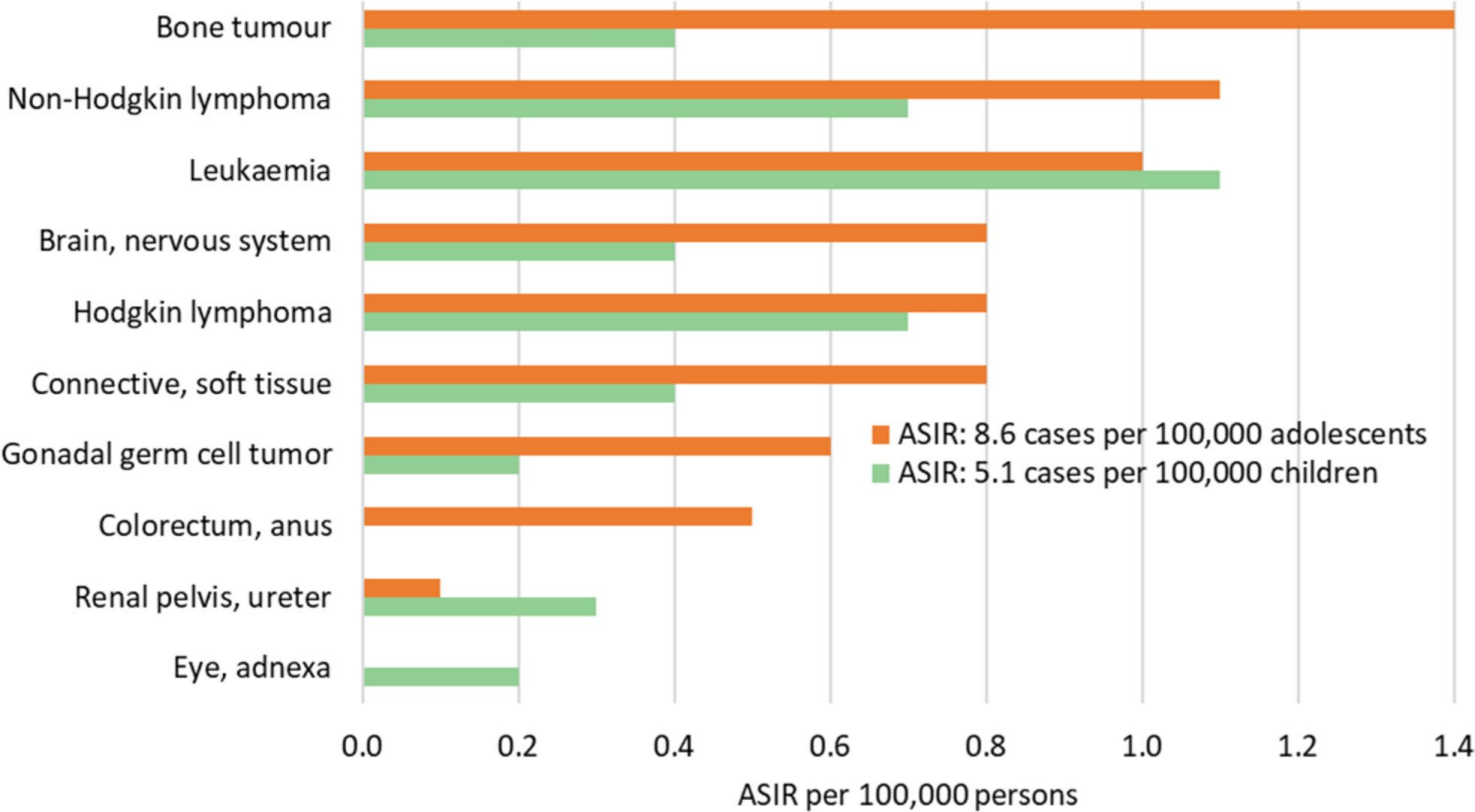
Age-Standardised Incidence Rates (ASIR) per 100,000 population by cancer site or type in children and adolescents, Khyber Pakhtunkhwa, Pakistan, 2020

**Fig. 5:**
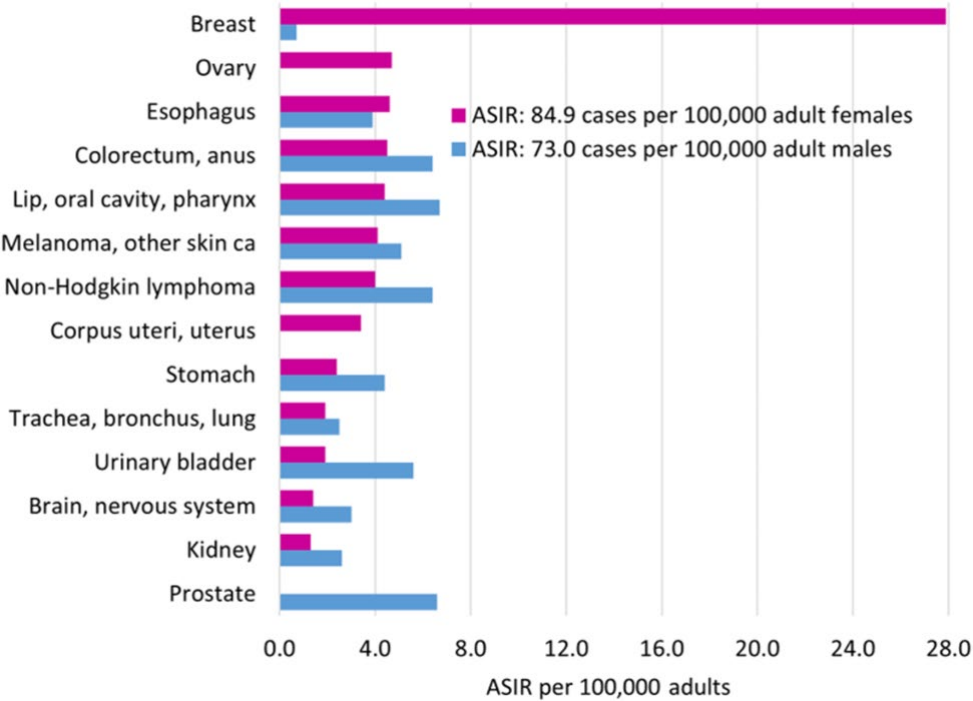
Age-Standardised Incidence Rates (ASIR) per 100,000 population by sex and cancer site or type in adults, Khyber Pakhtunkhwa, Pakistan, 2020

Figure 2 shows the population pyramid for the KP province in 2020. The population was young, with approximately 44% of individuals under the age of 15, 11% between the ages of 15 and 19, and 45% adults. There were 11,763 patients, nineteen of whom experienced multiple primaries. In all ages and both sexes, 11,782 new cancer cases were reported, with a slight preponderance in females (51.7%). Almost 7.4% of the cases were recorded in children, 3.0% in adolescents, and 89.6% in adults. Table 1 displays the statistics for the cancer site or type by age group and sex in children and adolescents, whereas Table 2 shows them in adults. The ASIR was calculated per 100,000 population; the overall ASIR was 49.8. The statistics for other categories were as follows: in children, 6.1 in boys and 4.0 in girls; in adolescents, 9.4 in males and 7.7 in females; and in adults, 73.0 in males and 84.9 in females (Fig. 3). According to the ICD-coding and ASIR, the top-ranking cancers in children were leukaemia (1.1), Hodgkin lymphoma (0.7), NHL (0.7), brain/nervous system (0.4), bone tumours (0.4), and connective/soft tissue tumours (0.4); in adolescents, bone tumours (1.4), NHL (1.1), leukaemia (1.0), brain/nervous system (0.8), Hodgkin lymphoma (0.8), and connective/soft tissue tumours (0.8); in adult females, cancers of the breast (27.9), ovary (4.7), oesophagus (4.6), colorectum/anus (4.5), and lip/oral cavity/pharynx (4.4); and in adult males, malignancies of the lip/oral cavity/pharynx (6.7), prostate (6.6), colorectum/anus (6.4), NHL (6.4), and urinary bladder (5.6) (Figs. 4 and 5).

**Table 1:** Age-Standardised Incidence Rates (ASIR) per 100,000 population by sex and cancer site or type in children and adolescents, Khyber Pakhtunkhwa, Pakistan, 2020

Incidence rate →	ICD-10-CM code	Children: girls				Children: boys				Adolescent: females				Adolescent: males			
Cancer site/type↓		N	%	Crude	ASIR	N	%	Crude	ASIR	N	%	Crude	ASIR	N	%	Crude	ASIR
Lip	C00	0	0.0	0.0	0.0	1	0.2	0.0	0.0	0	0.0	0.0	0.0	0	0.0	0.0	0.0
Tongue	C01-C02	0	0.0	0.0	0.0	0	0.0	0.0	0.0	0	0.0	0.0	0.0	0	0.0	0.0	0.0
Mouth	C03-C06	0	0.0	0.0	0.0	0	0.0	0.0	0.0	3	2.0	0.2	0.2	1	0.5	0.0	0.0
Salivary glands	C07-C08	2	0.6	0.0	0.0	0	0.0	0.0	0.0	0	0.0	0.0	0.0	0	0.0	0.0	0.0
Tonsil	C10	0	0.0	0.0	0.0	0	0.0	0.0	0.0	0	0.0	0.0	0.0	0	0.0	0.0	0.0
Nasopharynx	C11	1	0.3	0.0	0.0	4	0.7	0.0	0.0	2	1.3	0.1	0.1	7	3.5	0.3	0.3
Hypopharynx	C12-C13	0	0.0	0.0	0.0	1	0.2	0.0	0.0	0	0.0	0.0	0.0	1	0.5	0.0	0.0
Pharynx	C14	0	0.0	0.0	0.0	0	0.0	0.0	0.0	1	0.7	0.1	0.1	0	0.0	0.0	0.0
Esophagus	C15	0	0.0	0.0	0.0	0	0.0	0.0	0.0	0	0.0	0.0	0.0	1	0.5	0.0	0.0
Stomach	C16	0	0.0	0.0	0.0	1	0.2	0.0	0.0	3	2.0	0.2	0.2	1	0.5	0.0	0.0
Small intestine	C17	0	0.0	0.0	0.0	0	0.0	0.0	0.0	0	0.0	0.0	0.0	0	0.0	0.0	0.0
Colon	C18	1	0.3	0.0	0.0	2	0.4	0.0	0.0	2	1.3	0.1	0.1	2	1.0	0.1	0.1
Rectum	C19-C20	3	0.9	0.0	0.0	5	0.9	0.1	0.1	5	3.3	0.3	0.3	8	4.0	0.4	0.4
Anus	C21	1	0.3	0.0	0.0	1	0.2	0.0	0.0	0	0.0	0.0	0.0	3	1.5	0.1	0.1
Liver	C22	3	0.9	0.0	0.0	4	0.7	0.0	0.0	2	1.3	0.1	0.1	1	0.5	0.0	0.0
Gall bladder, etc	C23-C24	0	0.0	0.0	0.0	0	0.0	0.0	0.0	1	0.7	0.1	0.1	0	0.0	0.0	0.0
Pancreas	C25	1	0.3	0.0	0.0	0	0.0	0.0	0.0	0	0.0	0.0	0.0	0	0.0	0.0	0.0
Nose, sinuses, etc	C30-C31	2	0.6	0.0	0.0	2	0.4	0.0	0.0	3	2.0	0.2	0.2	0	0.0	0.0	0.0
Larynx	C32	0	0.0	0.0	0.0	0	0.0	0.0	0.0	0	0.0	0.0	0.0	0	0.0	0.0	0.0
Trachea, bronchus, lung	C33-C34	1	0.3	0.0	0.0	0	0.0	0.0	0.0	1	0.7	0.1	0.1	1	0.5	0.0	0.0
Other thoracic organs	C37-C39	0	0.0	0.0	0.0	1	0.2	0.0	0.0	0	0.0	0.0	0.0	0	0.0	0.0	0.0
Bone	C40-C41	24	7.4	0.3	0.3	47	8.5	0.5	0.5	20	13.1	1.0	1.0	39	19.6	1.9	1.9
Melanoma of the skin	C43	2	0.6	0.0	0.0	1	0.2	0.0	0.0	1	0.7	0.1	0.1	0	0.0	0.0	0.0
Other skin	C44	1	0.3	0.0	0.0	0	0.0	0.0	0.0	3	2.0	0.2	0.2	1	0.5	0.0	0.0
Mesothelioma	C45	0	0.0	0.0	0.0	1	0.2	0.0	0.0	0	0.0	0.0	0.0	0	0.0	0.0	0.0
Connective, soft tissue	C47,C49	33	10.2	0.4	0.4	33	6.0	0.4	0.4	16	10.5	0.8	0.8	15	7.5	0.7	0.7
Breast	C50	0	0.0	0.0	0.0	0	0.0	0.0	0.0	6	3.9	0.3	0.3	1	0.5	0.0	0.0
Vulva	C51	0	0.0	0.0	0.0	-	-	-	-	0	0.0	0.0	0.0	-	-	-	-
Vagina	C52	0	0.0	0.0	0.0	-	-	-	-	0	0.0	0.0	0.0	-	-	-	-
Cervix uteri	C53	0	0.0	0.0	0.0	-	-	-	-	1	0.7	0.1	0.1	-	-	-	-
Corpus uteri	C54	0	0.0	0.0	0.0	-	-	-	-	0	0.0	0.0	0.0	-	-	-	-
Uterus unspecified	C55	0	0.0	0.0	0.0	-	-	-	-	0	0.0	0.0	0.0	-	-	-	-
Ovary	C56	13	4.0	0.2	0.2	-	-	-	-	15	9.8	0.8	0.8	-	-	-	-
Other female genital organs	C57	1	0.3	0.0	0.0	-	-	-	-	0	0.0	0.0	0.0	-	-	-	-
Placenta	C58	0	0.0	0.0	0.0	-	-	-	-	0	0.0	0.0	0.0	-	-	-	-
Penis	C60	-	-	-	-	0	0.0	0.0	0.0	-	-	-	-	0	0.0	0.0	0.0
Prostate	C61	-	-	-	-	1	0.2	0.0	0.0	-	-	-	-	0	0.0	0.0	0.0
Testis	C62	-	-	-	-	10	1.8	0.1	0.1	-	-	-	-	8	4.0	0.4	0.4
Other male genital organs	C63	-	-	-	-	1	0.2	0.0	0.0	-	-	-	-	0	0.0	0.0	0.0
Kidney	C64	28	8.7	0.3	0.4	29	5.3	0.3	0.3	1	0.7	0.1	0.1	4	2.0	0.2	0.2
Renal pelvis	C65	1	0.3	0.0	0.0	1	0.2	0.0	0.0	0	0.0	0.0	0.0	0	0.0	0.0	0.0
Ureter	C66	0	0.0	0.0	0.0	0	0.0	0.0	0.0	0	0.0	0.0	0.0	0	0.0	0.0	0.0
Urinary bladder	C67	0	0.0	0.0	0.0	4	0.7	0.0	0.0	1	0.7	0.1	0.1	0	0.0	0.0	0.0
Other urinary organs	C68	0	0.0	0.0	0.0	0	0.0	0.0	0.0	0	0.0	0.0	0.0	0	0.0	0.0	0.0
Eye	C69	16	5.0	0.2	0.2	21	3.8	0.2	0.3	1	0.7	0.1	0.1	0	0.0	0.0	0.0
Brain, nervous system	C70-C72	45	13.9	0.5	0.5	33	6.0	0.4	0.4	19	12.4	1.0	1.0	15	7.5	0.7	0.7
Thyroid	C73	4	1.2	0.0	0.0	1	0.2	0.0	0.0	5	3.3	0.3	0.3	5	2.5	0.2	0.2
Adrenal	C74	2	0.6	0.0	0.0	2	0.4	0.0	0.0	0	0.0	0.0	0.0	0	0.0	0.0	0.0
Other endocrine	C75	0	0.0	0.0	0.0	0	0.0	0.0	0.0	0	0.0	0.0	0.0	0	0.0	0.0	0.0
Hodgkin lymphoma	C81	33	10.2	0.4	0.4	89	16.2	1.0	1.0	15	9.8	0.8	0.8	16	8.0	0.8	0.8
Non-Hodgkin lymphoma	C82-C86, C88.4, C96	36	11.1	0.4	0.4	89	16.2	1.0	1.0	8	5.2	0.4	0.4	37	18.6	1.8	1.8
Immunoproliferative diseases	C88	0	0.0	0.0	0.0	0	0.0	0.0	0.0	0	0.0	0.0	0.0	0	0.0	0.0	0.0
Multiple myeloma	C90	0	0.0	0.0	0.0	0	0.0	0.0	0.0	0	0.0	0.0	0.0	0	0.0	0.0	0.0
Lymphoid leukaemia	C91	42	13.0	0.5	0.5	101	18.4	1.1	1.1	7	4.6	0.4	0.4	12	6.0	0.6	0.6
Myeloid leukaemia	C92-94	11	3.4	0.1	0.1	27	4.9	0.3	0.3	5	3.3	0.3	0.3	12	6.0	0.6	0.6
Leukaemia unspecified	C95	3	0.9	0.0	0.0	20	3.6	0.2	0.2	1	0.7	0.1	0.1	5	2.5	0.2	0.2
MDS^a^	MDS	0	0.0	0.0	0.0	0	0.0	0.0	0.0	0	0.0	0.0	0.0	0	0.0	0.0	0.0
MPD^b^	MPD	1	0.3	0.0	0.0	0	0.0	0.0	0.0	0	0.0	0.0	0.0	0	0.0	0.0	0.0
Other & unspecified	O&U	8	2.5	0.1	0.1	15	2.7	0.2	0.2	2	1.3	0.1	0.1	2	1.0	0.1	0.1
Benign CNS^c^	Benign CNS	4	1.2	0.0	0.1	2	0.4	0.0	0.0	3	2.0	0.2	0.2	1	0.5	0.0	0.0
All sites (total)		323	100.0	3.9	4.0	550	100.0	6.2	6.1	153	100.0	7.7	7.7	199	100.0	9.4	9.4

^a^*MDS* Myelodysplastic Syndrome

^b^*MPD* Myeloproliferative Disorder

^c^*CNS* Central Nervous System

**Table 2:** Age-Standardised Incidence Rates (ASIR) per 100,000 population by sex and cancer site or type in adults, Khyber Pakhtunkhwa, Pakistan, 2020

Incidence rate →	ICD-10-CM code	Adult: females				Adult: males			
Cancer site/type↓		N	%	Crude	ASIR	N	%	Crude	ASIR
Lip	C00	12	0.2	0.1	0.2	23	0.5	0.3	0.4
Tongue	C01-C02	56	1.0	0.6	0.9	62	1.3	0.7	1.0
Mouth	C03-C06	79	1.4	0.9	1.3	163	3.3	1.9	2.5
Salivary glands	C07-C08	33	0.6	0.4	0.5	33	0.7	0.4	0.5
Tonsil	C10	10	0.2	0.1	0.1	10	0.2	0.1	0.1
Nasopharynx	C11	15	0.3	0.2	0.2	78	1.6	0.9	1.1
Hypopharynx	C12-C13	72	1.3	0.8	1.1	71	1.4	0.8	1.0
Pharynx	C14	4	0.1	0.0	0.1	1	0.0	0.0	0.0
Esophagus	C15	276	4.9	3.1	4.6	251	5.1	2.9	3.9
Stomach	C16	155	2.8	1.8	2.4	284	5.8	3.3	4.4
Small intestine	C17	22	0.4	0.2	0.3	44	0.9	0.5	0.7
Colon	C18	126	2.2	1.4	2.0	182	3.7	2.1	2.6
Rectum	C19-C20	155	2.8	1.8	2.1	230	4.7	2.7	3.2
Anus	C21	24	0.4	0.3	0.4	41	0.8	0.5	0.6
Liver	C22	72	1.3	0.8	1.2	118	2.4	1.4	1.9
Gall bladder, etc	C23-C24	93	1.7	1.1	1.5	53	1.1	0.6	0.8
Pancreas	C25	51	0.9	0.6	0.9	82	1.7	1.0	1.3
Nose, sinuses, etc	C30-C31	28	0.5	0.3	0.5	23	0.5	0.3	0.3
Larynx	C32	22	0.4	0.2	0.3	105	2.1	1.2	1.6
Trachea, bronchus, lung	C33-C34	116	2.1	1.3	1.9	166	3.4	1.9	2.5
Other thoracic organs	C37-C39	10	0.2	0.1	0.2	10	0.2	0.1	0.2
Bone	C40-C41	40	0.7	0.5	0.5	55	1.1	0.6	0.6
Melanoma of the skin	C43	30	0.5	0.3	0.5	26	0.5	0.3	0.4
Other skin	C44	209	3.7	2.4	3.6	306	6.2	3.6	4.7
Mesothelioma	C45	27	0.5	0.3	0.5	28	0.6	0.3	0.4
Connective, soft tissue	C47,C49	72	1.3	0.8	0.9	119	2.4	1.4	1.6
Breast	C50	1953	34.8	22.1	27.9	47	1.0	0.5	0.7
Vulva	C51	15	0.3	0.2	0.3	-	-	-	-
Vagina	C52	11	0.2	0.1	0.2	-	-	-	-
Cervix uteri	C53	134	2.4	1.5	2.2	-	-	-	-
Corpus uteri	C54	95	1.7	1.1	1.6	-	-	-	-
Uterus unspecified	C55	102	1.8	1.2	1.7	-	-	-	-
Ovary	C56	306	5.4	3.5	4.7	-	-	-	-
Other female genital organs	C57	8	0.1	0.1	0.1	-	-	-	-
Placenta	C58	9	0.2	0.1	0.1	-	-	-	-
Penis	C60	-	-	-	-	1	0.0	0.0	0.0
Prostate	C61	-	-	-	-	403	8.2	4.7	6.6
Testis	C62	-	-	-	-	78	1.6	0.9	0.8
Other male genital organs	C63	-	-	-	-	0	0.0	0.0	0.0
Kidney	C64	89	1.6	1.0	1.3	173	3.5	2.0	2.6
Renal pelvis	C65	4	0.1	0.0	0.1	2	0.0	0.0	0.0
Ureter	C66	0	0.0	0.0	0.0	1	0.0	0.0	0.0
Urinary bladder	C67	107	1.9	1.2	1.9	358	7.2	4.2	5.6
Other urinary organs	C68	1	0.0	0.0	0.0	1	0.0	0.0	0.0
Eye	C69	11	0.2	0.1	0.2	18	0.4	0.2	0.3
Brain, nervous system	C70-C72	105	1.9	1.2	1.4	238	4.8	2.8	3.0
Thyroid	C73	143	2.5	1.6	2.0	40	0.8	0.5	0.5
Adrenal	C74	4	0.1	0.0	0.0	4	0.1	0.0	0.0
Hodgkin lymphoma	C81	38	0.7	0.4	0.4	99	2.0	1.2	1.3
Non-Hodgkin lymphoma	C82-C86, C88.4, C96	251	4.5	2.8	4.0	435	8.8	5.1	6.4
Immunoproliferative diseases	C88	1	0.0	0.0	0.0	2	0.0	0.0	0.0
Multiple myeloma	C90	36	0.6	0.4	0.6	58	1.2	0.7	0.9
Lymphoid leukaemia	C91	29	0.5	0.3	0.4	67	1.4	0.8	0.9
Myeloid leukaemia	C92-94	53	0.9	0.6	0.7	60	1.2	0.7	0.7
Leukaemia unspecified	C95	6	0.1	0.1	0.1	9	0.2	0.1	0.1
MDS^a^	MDS	0	0.0	0.0	0.0	0	0.0	0.0	0.0
MPD^b^	MPD	0	0.0	0.0	0.0	1	0.0	0.0	0.0
Other & unspecified	O&U	212	3.8	2.4	3.3	230	4.7	2.7	3.4
Benign CNS^c^	Other benign CNS	86	1.5	1.0	1.2	50	1.0	0.6	0.7
All sites (total)		5618	100.0	63.5	84.9	4939	100.0	57.8	73.0

^a^*MDS* Myelodysplastic Syndrome

^b^*MPD* Myeloproliferative Disorder

^c^*CNS* Central Nervous System

## Discussion

Among children, haematologic malignancies contributed significantly to the cancer burden, whereas in adolescents, bone tumours, haematologic malignancies, and cancers of the brain and nervous system predominated. Although the historical periods differed from one another, this pattern resembles that observed among the population of the district of Lahore in the province of Punjab, Pakistan [19].

In adult women, cancers of the breast, digestive tract (oesophagus, stomach, and colorectum/anus), reproductive organs, tobacco-related cancers (lip/oral cavity/pharynx and lower respiratory tract), skin (melanoma and non-melanoma), NHL, urinary bladder, brain/nervous system, and kidney were recorded in the top-ten cancers. Among adult men, cancers of the digestive system and tobacco-related cancers were most frequently diagnosed, followed by those of the prostate, NHL, urinary bladder, skin, brain/nervous system, and kidney. Apart from oesophageal and stomach cancers, they were comparable in frequency to those in Lahore, Punjab [19], even though the ethnic makeup of the two regions is different. Lahore is also the most populous of Punjab’s 36 districts and has a population of over 11 million people [19]. The results from these areas might be typical of Pakistan’s population. The Karachi Division, which has 16 million residents and is distantly situated in the south of the country, also showed similarities in the distribution of cancer [2].

The Globocan report on cancers in Afghanistan, to the immediate west of KP province, was reviewed [20]. Breast, cervix uteri, digestive system (lip/oral cavity, oesophagus, stomach, colorectum, and liver), brain/nervous system, lung, and leukaemia were among the top ten cancers diagnosed in women of all ages. In comparison, gastrointestinal tract, lung, brain/nervous system, and leukaemia were the most frequently diagnosed cancers in men.

It has been estimated that 13% of the Pakistani population uses smokeless tobacco products [21]. Naswar, a type of smokeless tobacco used either as a dry powder inhaled as a snuff or placed in the mouth as a wad, is consumed extensively in Pakistan and Afghanistan [22]. It is readily available in the community and does not fall under the tax net [21]. Naswar makes up over 60% of the tobacco consumed in Peshawar, the capital of KP [21]. In a study conducted in KP, the likelihood of developing oral cancer was 20-times higher in those who had ever used naswar compared to those who had never used it [Odds Ratio (OR) 21.2, 95% Confidence Interval (CI) 8.4–53.8] [22]. In another study, the pooled estimate (OR) for oral cancer among ever-users of naswar compared to never-users in KP was calculated to be 18.3, which was again high (95% CI, 8.7–38.5) [23]. The meta-odds ratio for any oral lesions with a potential for malignant transformation associated with the use of any smokeless tobacco products was also extremely high, at 15.5 (95% CI, 9.9–24.2) in another systematic review conducted in South Asia [24].

Basal cell carcinoma was the most prevalent malignant eyelid tumor, followed by squamous cell carcinoma, according to a study of 222 individuals with malignant eyelid tumors from a major teaching hospital in Peshawar [25]. In addition, another institution-based study from Peshawar reviewed 986 cases of histologically proven oral and maxillofacial cancer and showed that squamous cell carcinoma was the most common diagnosis [26].

Of all the cancers recorded, the incidence of breast cancer was the highest. However, there is a dearth of information related to factors implicated in the aetiology of various cancers, including breast cancer, in the northwest region. Further, there is no consensus on a single factor implicated in the carcinogenesis of breast cancer [19]. However, factors such as early age at menarche, single marital status, nulliparity, delayed first full-term pregnancy, the use of oral contraceptives, delayed menopause, a family history of breast cancer, and a high body mass index could be linked to an increased risk of developing the disease [19]. A recent study has shown that BRCA1, BRCA2, and TP53 selected single nucleotide polymorphism risk alleles and risk allele-containing genotypes displayed a significant association (*p* < 0.05) with breast cancer risk in the Pashtun population [27]. A study conducted in 2015 suggested that the presence of GSTM1 and/or GSTT1 null genotypes, along with variant alleles of CYP1A1, might be the risk alleles for oral cancer in Pashtuns [28]. Yet another study conducted in 2015 on the whole genome sequencing of the Pashtun population in the northwest showed that a single nucleotide variation representing Ser217Leu in the ELAC2 gene (rs4792311) was also found and was implicated in genetic susceptibility to hereditary prostate cancer [29, 30]. The findings of another study conducted in 2014 suggested that the presence of the C allele could be a risk factor for esophageal cancer in the Pashtun population [31].

In terms of infectious agents, a study on the Hepatitis-C Virus (HCV) in KP indicated that genotype 3a was the predominant genotype (48%) and that the province had a high rate of HCV cirrhosis [32]. It is also known that those with HCV cirrhosis are more likely to develop liver cancer [33]. Three percent of oesophageal squamous cell carcinomas were found to have the human papillomavirus, according to another study from Peshawar [34]. Given that infectious agents have been identified significantly in KP, it would be worthwhile considering large-scale studies to explore further the aetiology of these diseases in KP.

## Conclusions

The number of cancer cases found among the inhabitants of KP in northwest Pakistan is substantial, according to this preliminary assessment. However, it is currently unclear what proportion of all malignancies these cases represent. To report the disease effectively and accurately, various stakeholders will need to work together on an ongoing basis in cancer registration. Achieving this goal becomes both desirable and doable if provincial and federal governments show commitment to developing a robust and well-resourced national public health system.

## Data Availability

The datasets generated and/or analysed during the current study are not publicly available because data have been collected from different centres and collated results presented. These centres have not allowed us to make the information available publicly, but the data are available from the corresponding author on reasonable request.

## Abbreviations

KP: Khyber Pakhtunkhwa
SKMCH&RC: Shaukat Khanum Memorial Cancer Hospital and Research Centre
ASIR: Age-Standardised Incidence Rate
NHL: Non-Hodgkin Lymphoma
FATA: Federally Administered Tribal Areas
PAEC: Pakistan Atomic Energy Commission
ICD-10-CM: The International Classification of Diseases, Tenth Revision, Clinical Modification
ICD-O-3: The International Classification of Diseases for Oncology, third edition
OR: Odds Ratio
CI: Confidence Interval
HCV: Hepatitis C Virus
IRB: Institutional Review Board
OHRP: Office for Human Research Protections
MDS: Myelodysplastic Syndrome
MPD: Myeloproliferative Disorder
CNS: Central Nervous System
FB: Farhana Badar
MS: Muhammad Sohaib
SM: Shahid Mahmood
ORC: Omar Rasheed Chughtai
FS: Faisal Sultan
MAY: Muhammed Aasim Yusuf
