# Correction to: Cancer incidence in Khyber Pakhtunkhwa, Pakistan, 2020

**DOI:** 10.1186/s12889-023-16888-x

**Published:** 2023-10-11

**Authors:** Farhana Badar, Muhammad Sohaib, Shahid Mahmood, Omar Rasheed Chughtai, Faisal Sultan, Muhammed Aasim Yusuf

**Affiliations:** 1 Cancer Registry and Clinical Data Management, Shaukat Khanum Memorial Cancer Hospital and Research Centre, 7-A, Block R-3, M. A. Johar Town, Lahore, 54782 Pakistan; 2 Nuclear Medicine & Oncology Division, Pakistan Atomic Energy Commission, Islamabad, Pakistan; 3 Chughtai Lab, Lahore, Pakistan; 4 Shaukat Khanum Memorial Cancer Hospital and Research Centre, Lahore, Pakistan

**Correction to**: ***BMC Public Health*23, 1785 (2023)**

10.1186/s12889-023-16686-5

The original publication of this article contained an incorrect version of Fig. 2. The order of the values (on opposite sides of the graphs) were reversed. The incorrect and correct version are shown in this correction article. The original article has been updated.

**Figure 1:**
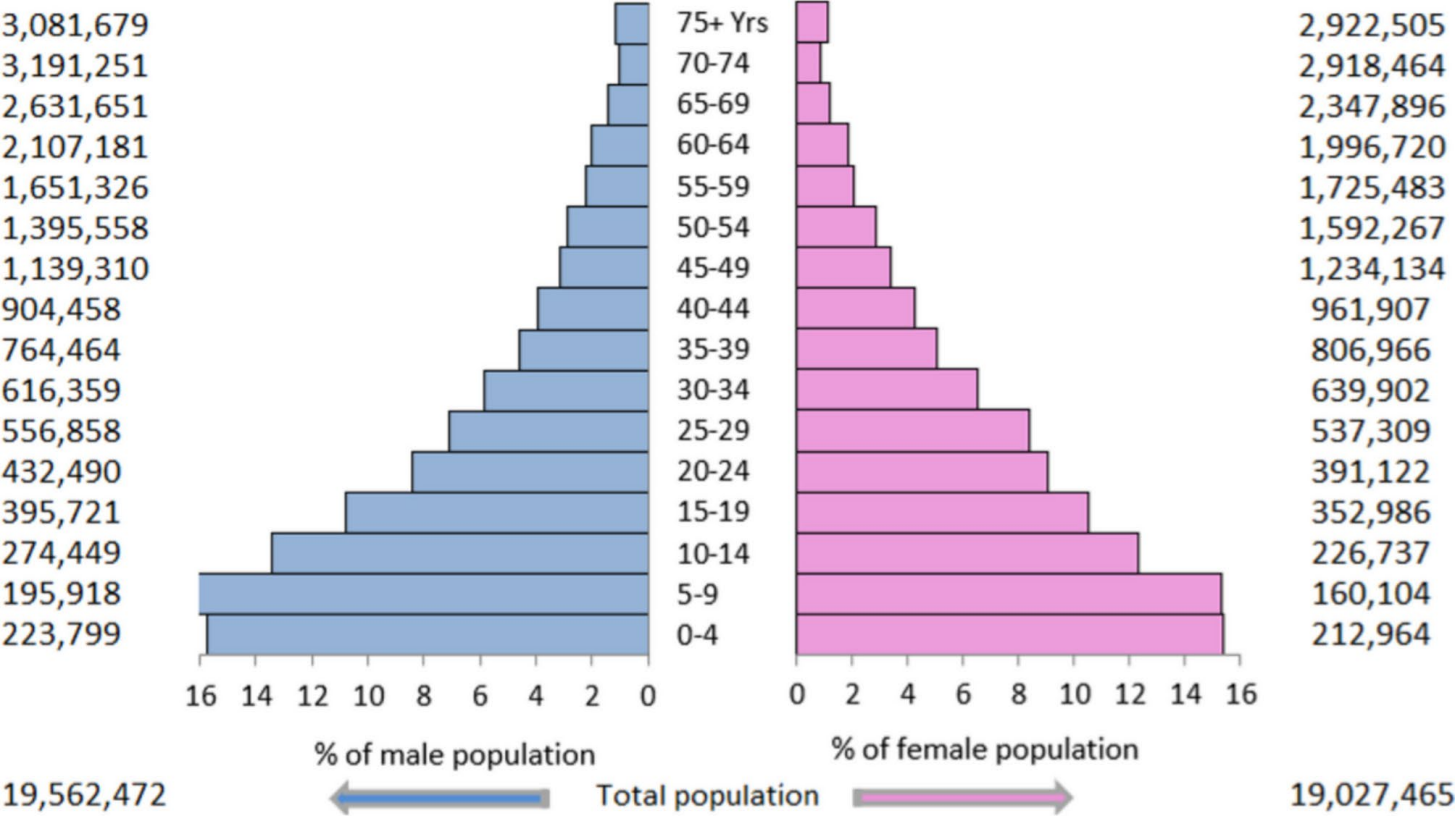
**Incorrect version of Fig. 2**

**Figure 2:**
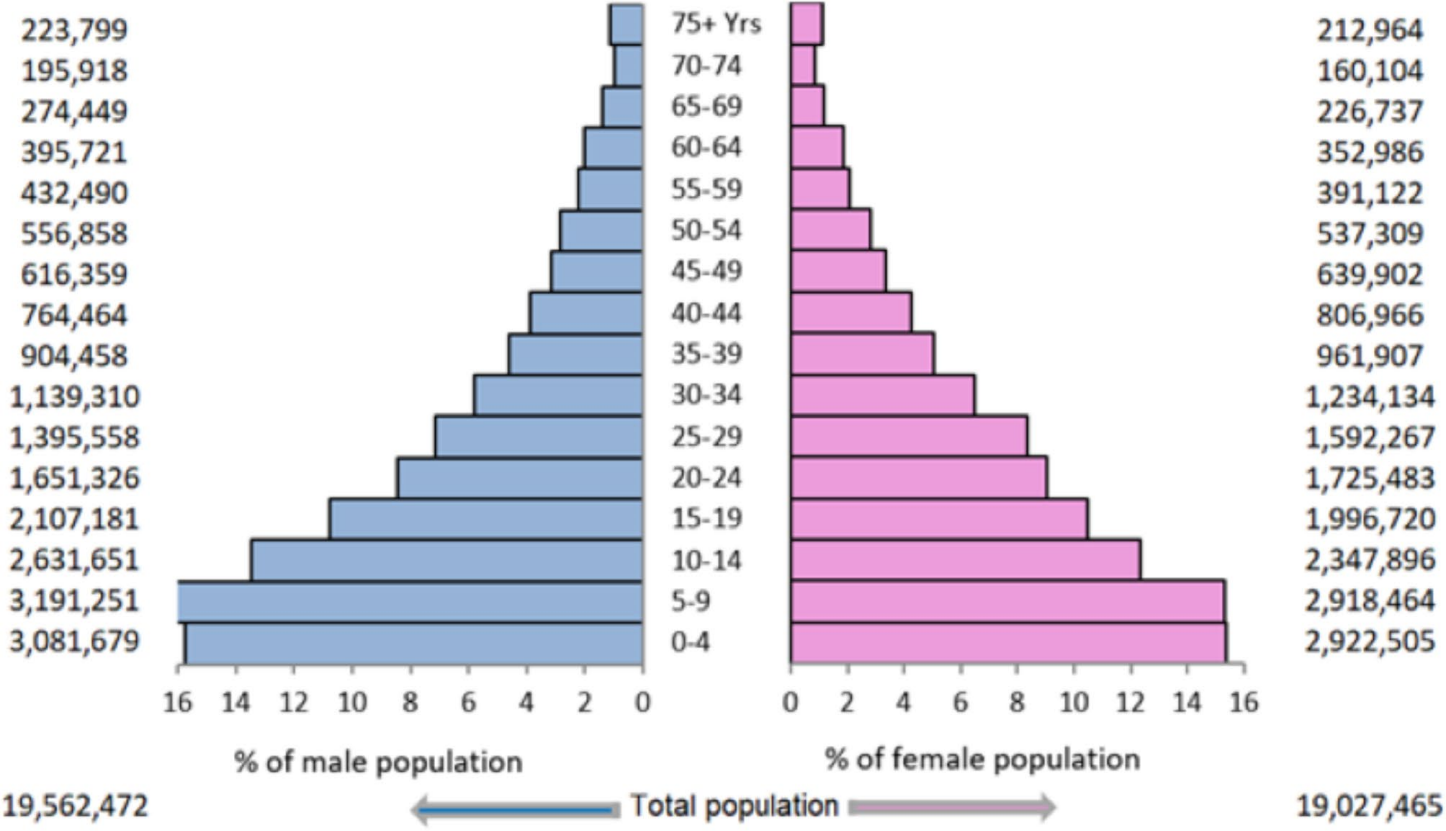
**Correct version of Fig. 2**

